# Supplementary material for: Platelets Modulate Leukocyte Population Composition Within Perivascular Adipose Tissue
Source: Int J Mol Sci. 2025 Feb 14;26(4):1625. doi: 10.3390/ijms26041625 (PMC11855773; doi:10.3390/ijms26041625)
Supplement: Supplementary file 1 [file ijms-26-01625-s001.zip › MDPI Supplementary Material_AC_010725.pdf]

International Journal of
Molecular Sciences

MDPI

Supplementary Material

Platelets Modulate Leukocyte Population Composition within Perivascular Adipose Tissue

Adam Corken [1,2], Tiffany Weinkopff [3], Elizabeth C. Wahl [2], James D. Sikes [2] and Keshari M. Thakali [1,2,*]

1 Department of Pediatrics, University of Arkansas for Medical Sciences, Little Rock, AR 72202, USA; alcorken@uams.edu
2 Arkansas Children's Research Institute, Little Rock, AR 72202, USA; wahlec@archildrens.org (E.C.W.); sikesjd@archildrens.org (J.D.S.)
3 Department of Microbiology and Immunology, University of Arkansas for Medical Sciences, Little Rock, AR 72205, USA; tsweinkopff@uams.edu
* Correspondence: kmthakali@uams.edu

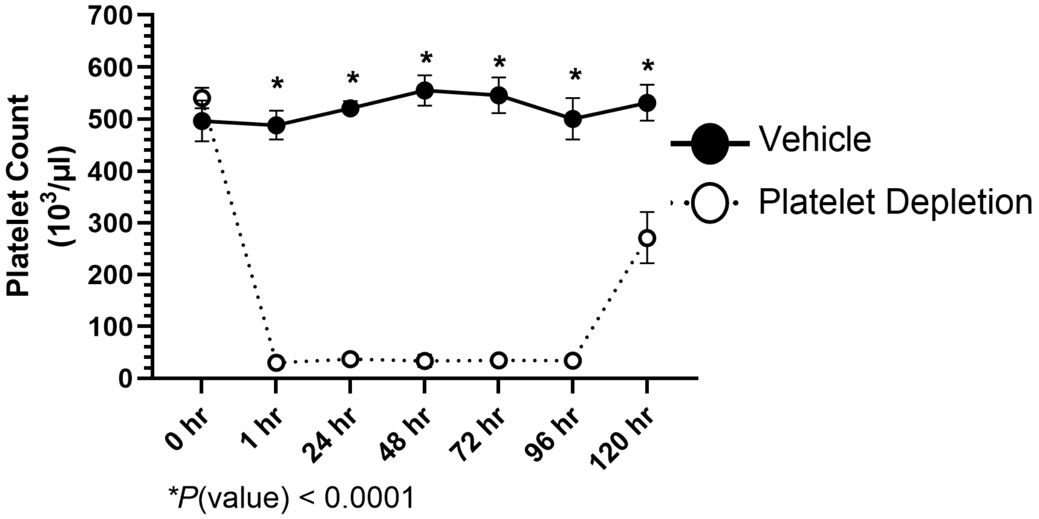

Supplementary Figure S1. The efficacy of the platelet depleting antibody was evaluated by quantifying platelet counts before and after antibody treatment at the specified intervals. The antibody treatment was referenced to a vehicle control cohort (PBS). Over the duration of the evaluation window the antibody treatment significantly reduced circulating platelet counts when compared to vehicle. Clinical thrombocytopenia is denoted as a reduction in platelet counts 10 % ≥ of normal and as such the antibody treatment successfully recapitulated a thrombocytopenic phenotype for 4 days with platelet counts beginning to normalize around day 5. N = 5.

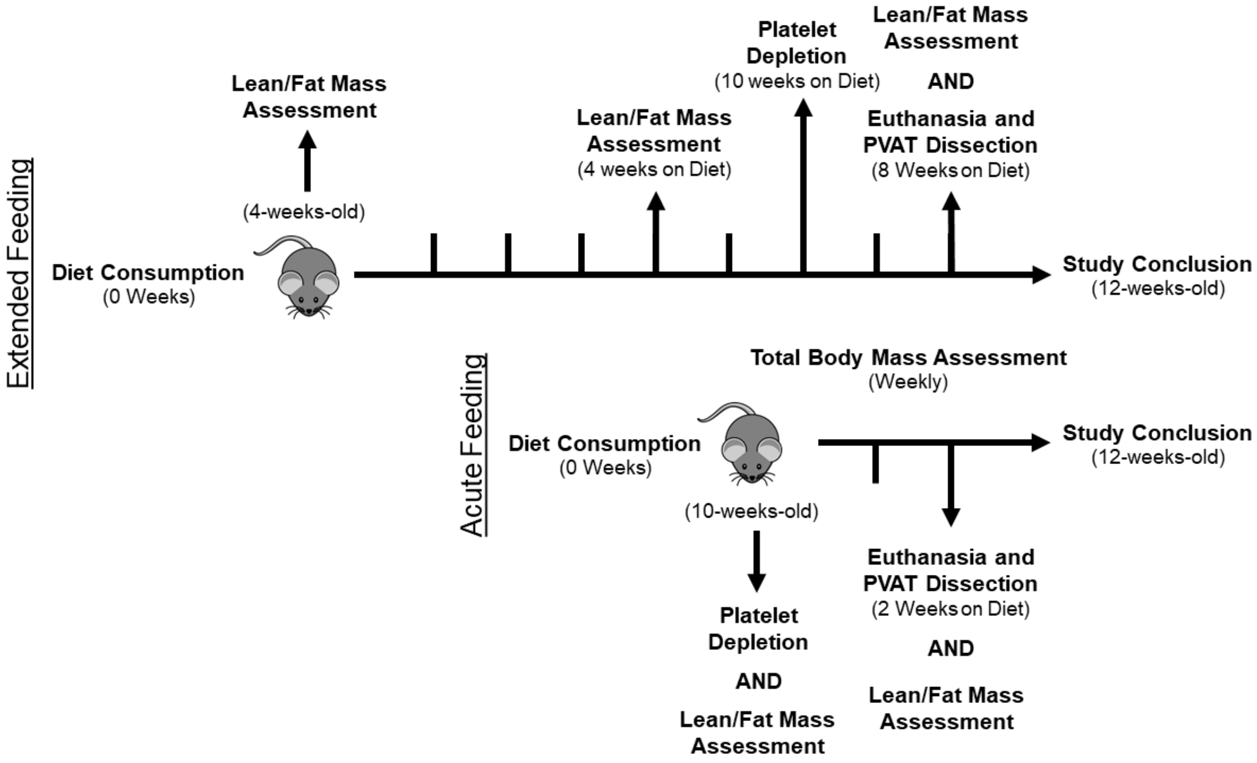

Supplementary Figure S2. A diagram illustrating the age of each mouse cohort at the time of dietary intervention as well at the study's conclusion. Additionally, the timing of mass assessments and vehicle or platelet depleting treatment.

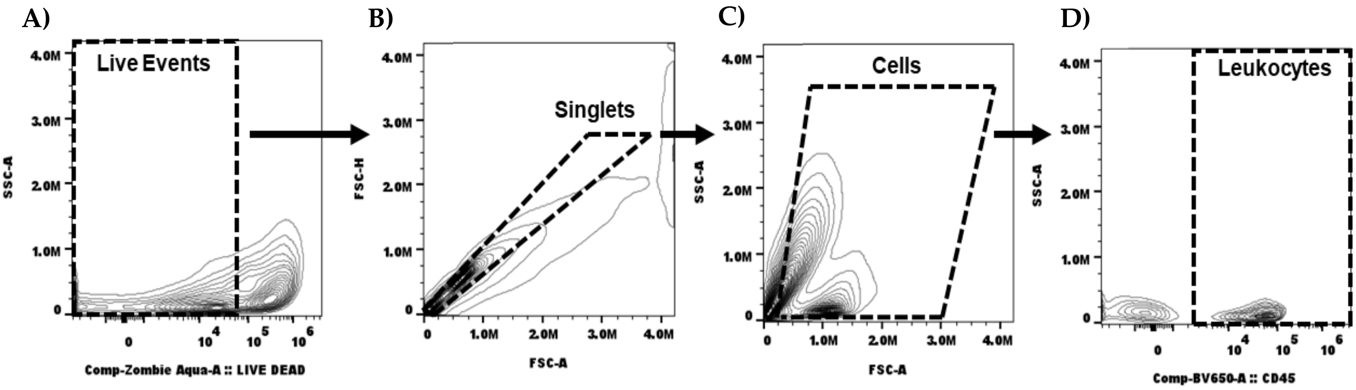

Supplementary Figure S3. A) The determination of the leukocyte population within PVAT began first by excluding all Zombie-Aqua+ non-viable events, followed by the selection of **B)** singlet events and lastly by gating onto events within the **C)** cellular forward and side scatter profile to exclude microparticle events, etc.. **D)** After the establishment of the "cell" population within the tissue sample, the leukocyte class was established by selection of the CD45+ population of cellular events.

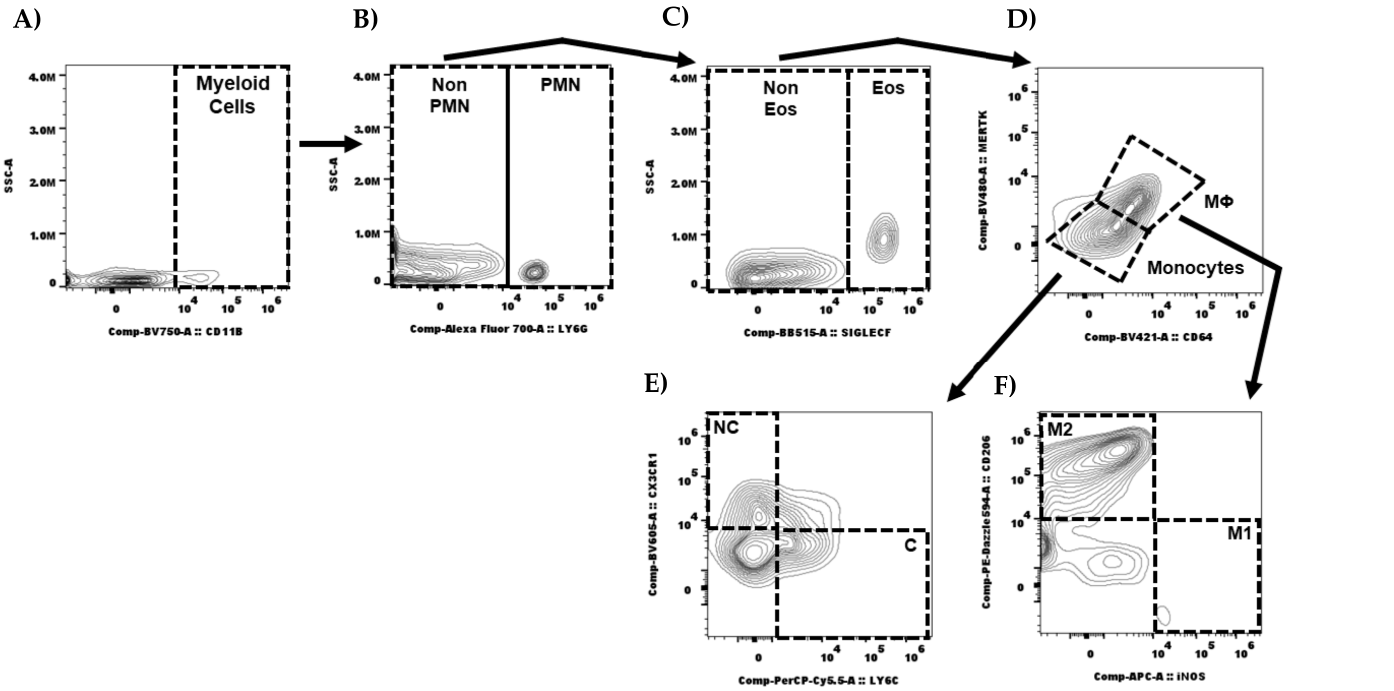

Supplementary Figure S4. A) The myeloid population of leukocyte as well as the visualization of its various subclasses began with the establishment of the myeloid class of cells as indicated by CD11b+ fluorescence. **B)** After establishing the myeloid cell population, the neutrophil (PMN) class was determined by Ly6G+ fluorescence. **C)** Within the remaining non-neutrophil events, eosinophils were next determined by Siglec-F+ fluorescence. **D)** The remaining non-eosinophil population was utilized to determine the monocyte and macrophage (MΦ) populations using MERTK and CD64. **E)** Classical (C) and nonclassical (NC) monocyte subclasses were determined using CXC3CR1 and Ly6C fluorescence. **F)** The macrophage M1 and M2 subclasses were determined via iNOS and CD206 fluorescence.

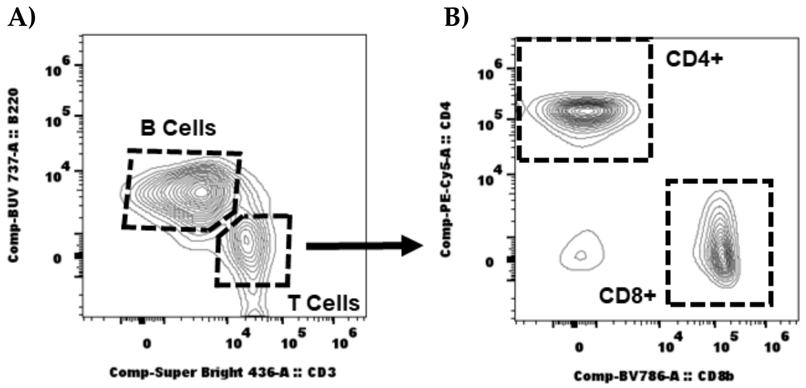

Supplementary Figure S5. A) Utilizing the non-myeloid population, the delineation between the B and T cell lymphocyte classes was established based on B220 and CD3 fluorescence. **B)** Further categorization of T cells into CD4+ and CD8+ subclasses was determined based on fluorescence of each marker.

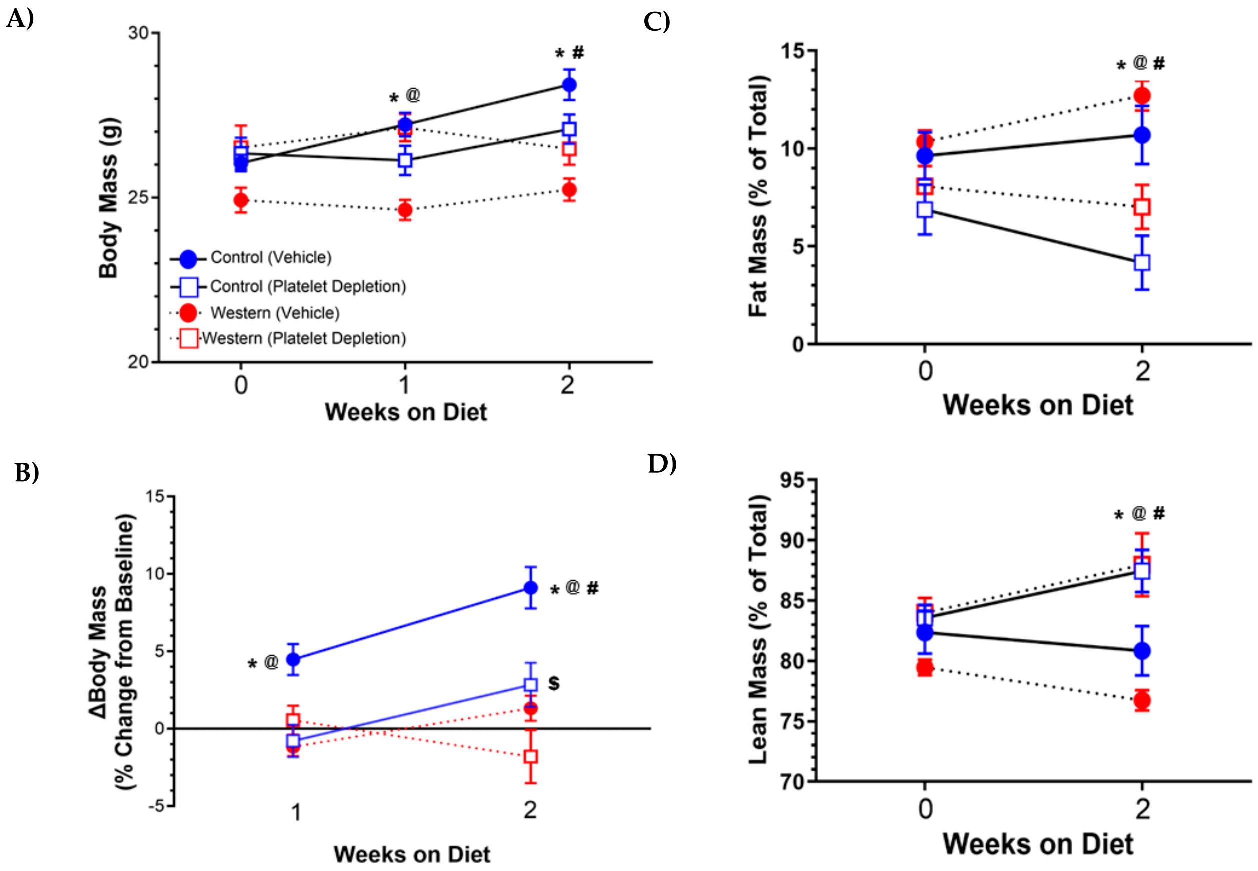

Supplementary Figure S6. Acute feeding of a Control and Western diet, coupled with platelet depleting antibody or vehicle treatment. **A)** Small but significant body mass changes accrued over 2 weeks of feeding with the Control diet group gaining more mass than Western fed animals. *Control vs Western *P*(value) < 0.05. @Western vs Western (Antibody) *P*(value) < 0.05. #Control vs Western (Antibody) *P*(value) < 0.05. **B)** Visualization of the percent change in total mass from baseline likewise showed the Control diet group having the highest rate of mass change at 1 and 2 weeks post diet intervention *Control vs Control (Antibody) *P*(value) < 0.05. @Control (Antibody) vs Western *P*(value) < 0.05. #Control vs Western (Antibody) *P*(value) < 0.05. $Control (Antibody) vs Western (Antibody) *P*(value) < 0.05. **C)** Reduced fat mass accumulation was associated with platelet depletion. *Control vs Control (Antibody) *P*(value) < 0.05. @Control (Antibody) vs Western *P*(value) < 0.05. #Western vs Western (Antibody) *P*(value) < 0.05. **D)** Increased lean mass accumulation was associated with platelet depletion. *Control vs Control (Antibody) *P*(value) < 0.05. @Control vs Western (Antibody) *P*(value) < 0.05. #Western vs Western (Antibody) *P*(value) < 0.05. Control N = 9. Control (Antibody) N = 8. Western N = 10. Western (Antibody) N = 8.

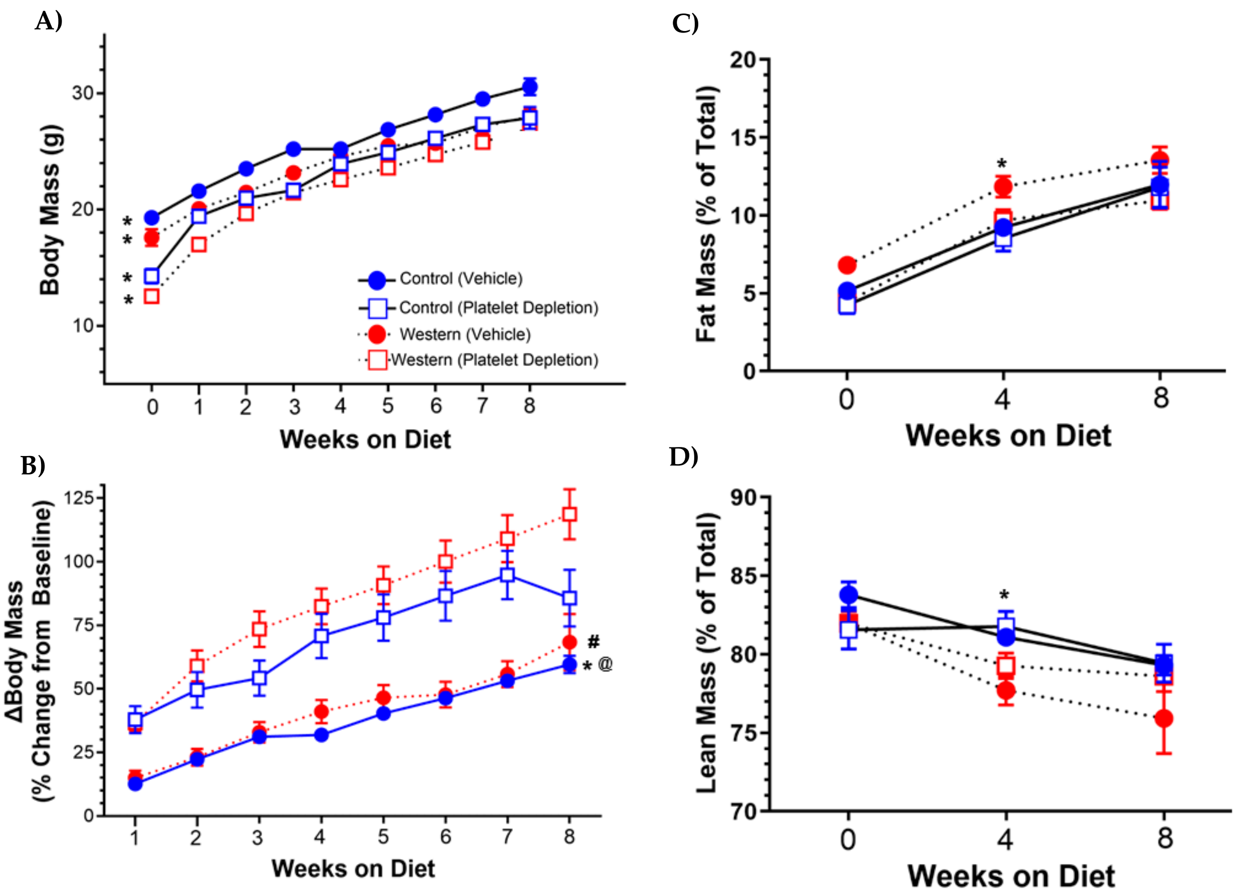

Supplementary Figure S7. Extended (8 week) feeding of a Control and Western diet in conjunction with platelet depleting antibody treatments. **A)** Significant mass differences were observed between all groups throughout the feeding window, but it should be noted that these differences were observed prior to the administration of diet. **P*(value) < 0.05 in comparison to all other groups. **B)** Viewing mass gain in relation to baseline values revealed platelet depleted groups had enhanced accumulation relative to those treated with vehicle. *Control vs Control (Antibody) *P*(value) < 0.05. [@]Control vs Western (Antibody) *P*(value) < 0.05. [#]Western vs Western (Antibody) *P*(value) < 0.05. **C)** The percentage and rate of fat mass accumulation with extended feeding was similar for all groups with the exception of the Western diet (Vehicle) group having significantly more fat mass than the Control diet (Antibody) group at 4 weeks of feeding. **P*(value) < 0.05. **D)** Inversely the percentage and rate of lean mass depletion was comparable among all groups with only exception again being the Western diet (Vehicle) cohort which contained significantly less lean mass relative to Control diet (Antibody) at 4 weeks of feeding. **P*(value) < 0.05. Control N = 9. Control (Antibody) N = 10. Western N = 10. Western (Antibody) N = 10.
